# Predicting Next‐Day Passive Suicidal Ideation in At‐Risk Youth

**DOI:** 10.1111/sltb.70124

**Published:** 2026-07-02

**Authors:** Shane Kentopp, Luke Francisco, Megan Chen, Ambuj Tewari, Ewa Czyz

**Affiliations:** ^1^ Department of Internal Medicine The University of Kansas School of Medicine Kansas City Kansas USA; ^2^ Department of Statistics University of Michigan Ann Arbor Michigan USA; ^3^ Department of Psychiatry University of Michigan Ann Arbor Michigan USA; ^4^ Department of Electrical Engineering and Computer Science University of Michigan Ann Arbor Michigan USA

**Keywords:** adolescents, ecological momentary assessment, intensive longitudinal data, machine learning, passive suicidal ideation, suicide prevention

## Abstract

**Introduction:**

Passive suicidal ideation (SI) is a well‐established risk factor for suicidal behavior but has received less attention than active SI. Although recent work has leveraged intensive longitudinal data and machine learning (ML) to forecast short‐term risk for active SI, passive SI remains understudied as a prediction target.

**Methods:**

Seventy‐eight psychiatrically hospitalized youth (ages 13–17 years) completed baseline assessments and daily ratings of risk and protective factors for 28 days post‐discharge. Multiple ML models were trained to predict the presence of next‐day passive SI. Models with and without baseline variables were compared to assess the relative predictive value of time‐varying versus baseline features.

**Results:**

ML models predicted next‐day passive SI with high accuracy (AUC = 0.90). The strongest predictors were within‐person 7‐day moving averages of passive SI duration and frequency. Including baseline variables had negligible performance impact, even during initial days post‐discharge.

**Conclusions:**

Short‐term passive SI remains an underutilized but important target for suicide prevention. Forecasting next‐day passive SI using ML is feasible and highly accurate. Within‐person, time‐varying features outperformed baseline factors, even in early days post‐discharge. Additional research on SI facets, such as duration, is needed. Integrating passive SI into personalized intervention frameworks may enhance the precision of suicide prevention efforts.

## 1 Introduction

Suicidal thoughts and behavior constitute a significant public health problem in the United States. Among youth, suicide is the second leading cause of death for those aged 10–14 years old and the third leading cause of death for youth aged 15–24 years old (Garnett and Curtin 2024). Suicidal ideation (SI), which refers to desiring to die or thinking about ending one's life, is among key predictors of future suicide attempts and deaths (Franklin et al. 2017; Hubers et al. 2016; Ribeiro et al. 2016), and is often a sign of significant distress. For youth specifically, the occurrence of SI is concerning for a suite of reasons. First, the sharpest increase in the number of suicide deaths across the lifespan occurs during the transition from adolescence to adulthood, and for many of those who have ever attempted suicide, the first onset of suicidal thoughts was during their youth (Cha et al. 2017). Further, adolescents report higher overall rates of SI compared to adults (Nock et al. 2008; Liu et al. 2020).

Suicidal thoughts include both passive ideation (a general wish to be dead) and active ideation (thoughts about taking one's life) (Liu et al. 2020). Active SI further lies on a continuum of severity, from general thoughts of killing self to suicidal thoughts with intent and plan. For this study, we refer to active SI as any thoughts of ending one's life, whereas passive SI includes wishes or desire to be dead without explicit thoughts of killing self. While existing suicide prevention literature has primarily focused on active SI, comparatively little attention has been given to passive SI (Liu et al. 2020). Nonetheless, both passive and active SI are significant risk factors for suicidal behavior, such as suicide attempts and deaths (Baca‐Garcia et al. 2011; Berman 2018; Liu et al. 2020). In recent years, there has been a surge of research using intensive longitudinal methods to characterize and predict SI in the near future (Kivelä et al. 2022; Kleiman et al. 2023; Sedano‐Capdevila et al. 2021). However, these studies have also generally emphasized active SI, and, to our knowledge, only a few studies have explicitly examined short‐term passive SI (e.g., Hamilton et al. 2024; Kivelä et al. 2024; Mandel et al. 2024; Smith et al. 2024). In order to advance suicide prevention efforts, there is an urgent need to improve our understanding of passive SI, particularly in everyday context.

Largely understudied (Baca‐Garcia et al. 2011), passive SI has been traditionally viewed as a less severe gateway to active ideation, and as a result, may be assumed to be less risky or impairing than active SI. Nonetheless, recent research has underscored that passive SI should be studied with the same urgency. A large meta‐analysis of both youth and adult samples found that the lifetime prevalence of passive SI was nearly 11% in epidemiological samples and as high as 50% in psychiatric populations (Liu et al. 2020). Even though passive SI is generally considered less severe, it has been implicated in numerous mental health outcomes and linked to future suicide attempts, and preliminarily, suicide deaths (Baca‐Garcia et al. 2011; Liu et al. 2020; May et al. 2015).

Leading ideation‐to‐action theories of suicide, including the Interpersonal Theory of Suicide (IPTS) and Three‐Step Theory of Suicide (3ST), emphasize the importance of distinguishing among forms of SI and provide a useful framework for understanding the differences between passive and active SI (Klonsky et al. 2021; Van Orden et al. 2010). Broadly, these theories posit that the emergence of SI and the progression from less severe ideation to more severe forms of ideation and, ultimately, behaviors are governed by partially distinct psychological processes and mechanisms. Within the IPTS framework, the presence of either thwarted belongingness or perceived burdensomeness is considered a proximal and sufficient cause of passive SI, whereas for active SI to develop, there must be a simultaneous presence of these two constructs and hopelessness about these states changing.

Consistent with these theoretical distinctions, psychometric evidence suggests that passive and active SI represent related but separable constructs. In a study by Wastler et al. (2022), exploratory factor analysis of the Self‐Injurious Thoughts and Behaviors Interview‐Revised scale revealed passive and active SI loading onto separate factors. Similarly, Steer et al. (1993) observed that the factor structure of the Beck Scale for Suicidal Ideation best supports latent constructs that distinguish between passive and active SI. Together, these theoretical perspectives and empirical findings emphasize that not all suicidal thoughts reflect the same underlying processes and underscore the importance of examining the phenomenology of passive SI as a meaningful clinical outcome distinct from active SI.

There has been notable research beginning to investigate passive SI as a time‐varying outcome using intensive longitudinal sampling. While some studies have focused exclusively on passive SI, others have examined both passive and active SI. For example, among college students, multiple forms of cognitive dysfunction (e.g., unbearability, attentional fixation, and rumination) were associated with concurrent passive SI while only rumination prospectively predicted passive SI (Mandel et al. 2024). In a network analysis study involving active duty service members and veterans, Smith et al. (2024) identified temporal group‐level networks, revealing passive SI at the next timepoint was predicted by alienation, burdensomeness, and hopelessness. Interestingly, active SI also predicted passive SI, suggesting these two SI states are interrelated over time. In another network analysis study with psychiatric inpatients, Kivelä et al. (2024) found positive contemporaneous associations between passive SI and factors such as sadness, loneliness, and burdensomeness. Temporally, acquired capability and hopelessness predicted passive ideation at the next timepoint, which, in turn, predicted active ideation. Together, these findings highlight distinct risk profiles of passive versus active SI, demonstrating that some risk factors are more unique to one form of ideation versus another.

To date, most studies of passive SI utilizing intensive longitudinal data have been conducted among adults, with only one study, to our knowledge, conducted with adolescents. Among adolescents recruited on social media, Hamilton et al. (2024) found that social media experiences may be dynamic and modifiable risk factors for SI. On days when teens reported more negative emotional responses to social media than their average, they also reported more passive death wish and active SI. Conversely, on days when teens experienced more positive social media experiences than usual, they were less likely to report both passive death wish and active SI that day. Collectively, these findings underscore a need to expand intensive longitudinal research on passive SI, as distinct from active SI, particularly in adolescents.

Recent work has adopted machine learning approaches to predicting the occurrence of active SI (Ballard et al. 2021; Bozzay et al. 2024; Choo et al. 2024; Czyz, Koo, et al. 2021; Czyz et al. 2023; Lei et al. 2023; Wang et al. 2021, 2024). Machine learning refers to computational approaches that allow algorithms to learn patterns from data to make predictions about future outcomes. Machine learning is useful for identifying complex relations among many factors while preventing overfitting (the tendency to exploit spurious patterns present in a sample of training data) through cross‐validation. This approach prioritizes generalizability by analyzing examples from a sample of training data and producing predictive models that optimize accuracy of predictions in new unseen data. As the field of suicide prevention moves towards implementing tailored interventions that can be delivered in real‐time (e.g., Nahum‐Shani et al. 2017), accurate and generalizable predictive models are critical. However, this line of research has focused primarily on active SI, with no previous studies to date using machine learning to make short‐term predictions about the occurrence of passive SI.

The current study extends existing research on the prediction of near‐term SI by predicting the occurrence of next‐day passive ideation, leveraging machine learning approaches, in a clinical sample of adolescents at elevated suicide risk. Given the scarcity of previous work in this area, this was an exploratory machine learning study. As such, its primary aim was prediction, rather than explanation (Yarkoni and Westfall 2017), and to generate hypotheses to be tested in future confirmatory research (Stebbins 2025). Thus, we did not posit any a priori hypotheses.

We investigated several research questions. First, we sought to establish how well various machine learning techniques can predict next‐day passive SI compared to the accuracy of active SI prediction from previous studies. These results represent an important accuracy benchmark for future research.

Second, we explored which predictors are most important for predicting passive SI among high‐risk adolescents. Guided by leading theories of suicide, we selected candidate predictors measured both cross‐sectionally at baseline and repeatedly at the daily level that were intended to map onto theoretically relevant constructs (e.g., hopelessness, perceived burdensomeness) in addition to other clinically meaningful variables (e.g., cognitive and affective variables) associated with SI. Thus, predictor selection was informed by broader theory, prior research, and clinical relevance. We expected our results to be directionally consistent, and we anticipated that generating a relative ranking of predictors would yield informative, theory‐relevant insights into which factors most strongly influence short‐term passive SI in this high‐risk window. However, we did not test any theoretical assertions in this exploratory investigation.

Third, we investigated the relative importance of cross‐sectional “baseline” data—collected at a single time point—compared to intensive longitudinal data collected through repeated assessments. Specifically, integrating cross‐sectional and intensive longitudinal data helps identify when, and for whom, SI is likely. This task is especially challenging in clinical settings, when forecasting risk for a new patient, for whom little or no previous assessment data is available. For example, the period immediately following discharge from a psychiatric admission is a vulnerable window and accurate predictions of SI during this time would be highly relevant. However, on the first day following discharge, the only data available for a new patient are those collected at baseline (i.e., during the admission). With the passage of time, repeated assessments can provide a richer dataset, but this process is expensive and time consuming. In order to develop efficient and scalable prevention efforts, it is important to consider the costs and benefits of collecting intensive longitudinal data. Finally, we analyzed whether the relative utility of these two different data types changed over time. Taken together, this intensive longitudinal study sought to address several key research gaps by leveraging machine learning models of varying complexity to predict near‐term passive SI among high‐risk adolescents.

## 2 Method

### 2.1 Participants

Participants included 78 youth, age 13–17 years old, admitted for psychiatric hospitalization due to suicide attempt in the last month or last‐week suicidal ideation with thoughts of method, intent, or plan. Exclusion criteria included cognitive impairment, altered mental status, transfer to medical unit or residential facility, no parent or guardian, or no access to a mobile phone. Participants were recruited between March 2019 and January 2020 as part of an intervention pilot study (see Czyz, King, et al. 2021). This study was approved by the Michigan Medicine Institutional Review Board (HUM00129173). After obtaining parental consent and adolescent assent, participants completed a baseline survey prior to discharge. Of the 94 individuals approached to participate in the study, 82 (87.2%) provided study consent/assent. A total of 80 participants completed a baseline survey prior to discharge and were enrolled in the study.

Daily surveys were completed for 4 weeks, beginning the first day after discharge. Daily surveys were sent automatically to participants via text message each evening. Participants were compensated a total of $222 for study participation, which included $4 for each daily survey. On‐call clinicians monitored daily survey responses and reached out to participants directly if they endorsed SI with intent/plan or a suicide attempt in the last 24 h. If any SI was reported, an automated message was displayed recommending participants seek support and providing emergency contact information. Daily surveys were completed at a rate of 74.2%.

### 2.2 Measures

#### 2.2.1 Baseline Measures

Participants completed a baseline survey assessing demographics and selected clinical risk factors for SI. Baseline feature selection was guided by the principles of model parsimony and clinical utility. We focused on nine variables that have been identified as robust predictors of near‐term suicidal thoughts and behavior. Although a more in‐depth history or thorough psychiatric interview could potentially provide helpful information, this is often not feasible to collect during the transition period from inpatient to outpatient care.

Demographics included biological sex, age, sexual minority status, and gender minority status. Sexual minority identity was defined as any self‐reported sexual orientation other than “straight.” Gender minority identity was defined as any self‐reported gender other than “cisgender male” or “cisgender female.” Baseline clinical risk factors included depressive symptoms, anxiety symptoms, hopelessness, multiple suicide attempt history, and lifetime frequency of non‐suicidal self‐injury. Depressive symptoms were assessed using the adolescent version of the Patient Health Questionnaire‐9 (PHQ‐9; Johnson et al. 2002) and anxiety symptoms were assessed using the Generalized Anxiety Disorder‐7 questionnaire (GAD‐7; Kroenke et al. 2007). Hopelessness was assessed using the 6‐item Brief Hopelessness Scale (Bolland et al. 2001), adapted from the Hopelessness Scale for Children (Kazdin et al. 1986). History of multiple suicide attempts was assessed via review of the participants' electronic medical records and was operationalized as a binary variable reflecting the presence of 2 or more lifetime suicide attempts. Lifetime NSSI frequency was assessed using a single item with a 7‐point Likert‐type scale with responses ranging from “once” to “more than 100 times.” Descriptive statistics for baseline variables are presented in Table 1 and a more thorough description of the sample can be found in Czyz, King, et al. (2021).

**TABLE 1:** Summary statistics for baseline predictors.

Predictor	Summary
Age	15.18 (1.35)
Female	52 (67.5%)
Heterosexual orientation	37 (48.1%)
Gender minority identity	6 (7.8%)
History of multiple suicide attempts	27 (35.1%)
Number of self‐injury instances, lifetime	3.47 (2.53)
Anxiety symptoms^a^	2.04 (0.78)
Depressive symptoms^b^	2.02 (0.59)
Hopelessness	2.67 (0.73)

*Note:* Mean first with SD in parentheses for continuous variables; count first with percentage in parentheses for categorical variables.

^a^ Youth GAD‐7 average item score.

^b^ Youth PHQ‐9 average item score.

#### 2.2.2 Daily Measures

##### 2.2.2.1 Affect

Three affective states were measured using items adapted from the Positive and Negative Affect Schedule for Children (PANAS‐C; Ebesutani et al. 2012). Participants were asked to rate how much they felt *happy*, *sad*, and *miserable* in the last 24 h on a 5‐point Likert‐type scale ranging from “Very slightly or not at all” to “Extremely.”

##### 2.2.2.2 Agitation

Agitation was measured using an item adopted from the Brief Agitation Measure (Ribeiro et al. 2011). Participants rated the extent to which they agreed with the statement, “I felt so stirred up inside I wanted to scream,” in the last 24 h on a 7‐point Likert‐type scale ranging from “Not at all” to “Very much.”

##### 2.2.2.3 Connectedness and Burdensomeness

Interpersonal connectedness with family and friends was measured using two items adapted from the Interpersonal Needs Questionnaire (INQ) (Van Orden et al. 2012). Participants rated the extent to which they agreed with two statements over the last 24 h: “I felt close to my family” and “I felt close to my friends.” We created a composite connectedness variable by taking the mean of these two items. Perceived burdensomeness was measured using an INQ item “The people in my life would be happier without me.” Responses to all items were made on a 7‐point Likert‐type scale ranging from “Not at all” to “Very much.”

##### 2.2.2.4 Hopelessness

Hopelessness was measured using a single item adapted from the Brief Hopelessness Scale (Bolland et al. 2001). Participants rated their agreement with the statement, “I see only bad things ahead of me, not good things,” on a 4‐point Likert‐type scale ranging from “Strongly disagree” to “Strongly agree.”

##### 2.2.2.5 Rumination and Worry

Rumination and worry were measured using separate items adapted from a previous ecological momentary assessment study by Kircanski et al. (2015). To assess rumination, participants rated their agreement in the last 24 h with the statement, “I was dwelling on my feelings and problems.” To assess worry, participants rated their agreement with the statement, “I was worried about things that could happen.” Responses to both items were made on a 7‐point Likert‐type scale ranging from “Not at all” to “Very much.”

##### 2.2.2.6 Self‐Efficacy

Self‐efficacy to resist suicide was assessed using an item adapted from the Self‐assessed Expectations of Suicide Risk Scale (Czyz et al. 2016). Participants responded to the question, “How confident are you that you will be able to keep yourself from attempting suicide?” on a scale from 0 (“not at all confident”) to 10 (“completely confident”).

##### 2.2.2.7 Facets of Suicidal Ideation

Active and passive SI were measured using items adapted from the Columbia‐Suicide Severity Rating Scale (Posner et al. 2011). To assess frequency of passive SI, participants were asked, “In the last 24 hours, how many times did you wish you were dead or that you could go to sleep and not wake up?” To assess frequency of active SI, participants were asked, “In the last 24 hours, how many times did you have thoughts of killing yourself?” Responses to both items ranged from 0 (“Not at all”) to 4 (“All the time”). To assess duration of active and passive SI, participants were then asked, “In the last 24 hours, how long did these thoughts last?” Responses ranged from 1 (“A few seconds or minutes”) to 5 (“More than 8 hours/continuous”).

### 2.3 Data Analysis Plan

#### 2.3.1 Feature Construction

We constructed three features from each construct of interest measured daily: two within‐person summary statistics capturing recent functioning (7‐day moving average and 7‐day moving standard deviation) and daily deviation from the 7‐day moving average capturing change from recent functioning. Models were tested using longer or shorter histories to construct these features, but gains in predictive ability diminished beyond 7 days. On days 1–6, the features were computed using all prior days. A binary variable indicating whether the person's time‐varying observations were missing on the current day was also constructed. In total, this yielded 53 predictors consisting of 9 initial (baseline) features, 14 × 3 features that were measured repeatedly (time‐varying), an indicator of missingness for the current day, and continuous time.

Using these predictors, we focused on predicting presence/absence of next‐day passive SI for each participant. Although passive SI frequency was originally measured on a continuous scale, as noted above, it was dichotomized into a binary variable for our predictive models. This decision was motivated by the clinical objective of the study to contribute to decision support tools for suicide prevention. A dichotomous variable allows for the calculation of performance metrics that are familiar to clinicians who are used to evaluating the utility of diagnostic tests (area under the ROC curve, sensitivity, specificity). From an intervention perspective, any presence of passive SI is clinically meaningful and would warrant a potential response, making the presence of these thoughts a natural decision threshold.

#### 2.3.2 Data Preparation

We eliminated 544 days of data owing to missing passive SI responses and another 16 days of data due to participants with no time‐varying data for computing cumulative predictors in the first days of the study. Thus, the analytic sample includes 1546 daily observations with information on predictors and the outcome of next‐day SI across 77 participants.

Due to the structure of the daily surveys, some values of passive SI duration were missing by design. To reduce participant burden, only frequency of passive SI was measured on days when participants endorsed presence of active SI (i.e., without assessing passive SI duration). For these cases (*n* = 601 out of 729 instances of passive ideation in the initial sample), we imputed passive SI duration from passive SI frequency with a linear regression model using complete cases (the other 128 instances of passive ideation). Both variables were log‐transformed prior to modeling. The imputation model was robust, explaining a significant portion of variance in duration (*R* ^2^ = 0.435, *b* = 0.861, *p* < 0.001). Our assumption of a linear relationship was supported by a moderate Pearson correlation (*r* = 0.43; adjusted for repeated measures) among the complete cases.

Among our final sample of 1546 daily observations, missingness rates for our predictors were < 1% for baseline values, < 1% for cumulative means, 6% for cumulative standard deviations, and 13% for daily deviations. We used mean imputation for all missing baseline predictors. For missing daily predictors, we used last observation carried forward (LOCF) within each participant for the cumulative means and cumulative standard deviations. This is a simple approach to imputation, but it allows us to easily fill in gaps for a small portion of observations using the most up‐to‐date measures. The remaining missing cumulative standard deviations were in the first daily observation for each subject and were replaced with 0 (since computing standard deviation requires more than one observation). Lastly, the within‐person daily deviations from the cumulative mean are dynamic indicators and not suited to LOCF imputation. For these, we imputed a value of zero. That is, we assume that the missing day's observation is equal to the moving average. After imputing missing data, we standardized our predictors to have mean of zero and standard deviation of one.

To assess whether our approach to missing data substantively impacted our findings, we replicated all analyses using only complete cases as a sensitivity analysis. The results of this sensitivity analysis were not meaningfully different from those reported below with respect to model performance and feature importance. Results from the complete case analysis are in Data S1.

#### 2.3.3 Predictive Modeling

To assess the accuracy with which passive SI can be predicted, we used a “persistence” model to establish a benchmark for comparison. This model predicts passive SI on a given day using the frequency of passive SI from the previous day. If the frequency of passive SI on the previous day was greater than zero, the persistence model predicted the presence of passive SI on the current day.

To investigate the relative importance of baseline and time‐varying predictors during the post‐discharge period, we trained a series of six machine learning models to predict next‐day SI: two versions (with baseline features, without baseline features) of three different algorithms (binomial elastic net, joint elastic net, random forest).

The first set of models was binomial logistic elastic net regressions, trained using the glmnet package (Friedman et al. 2010) in R to select the best regularization hyperparameters that maximized the area under the ROC curve (AUC). Next, we fit multinomial logistic elastic net models that predicted one of four classes constructed from all possible combinations of the presence/absence of passive/active SI. We call this our “joint” model because it jointly predicts passive and active ideation. This model can learn coefficient estimates unique to those who have passive ideation but not active (or vice versa). Although predicting active SI is not the focus of the current study, it is possible that modeling the joint probability distribution of passive and active SI may improve prediction of passive SI. Lastly, we fit a random forest model to investigate prediction with nonlinear machine learning methods. Random forests have previously shown promise predicting suicide‐related outcomes (van Mens et al. 2020; Walsh et al. 2018). We trained our model using the randomForest R package (Liaw and Wiener 2002) to select values for the number of trees in the forest (ntree) and number of predictors sampled for each decision rule (mtry) that maximized cross‐validated AUC.

For all predictive models, we used a blocked and nested cross‐validation strategy, which confers multiple advantages, with 5‐fold cross‐validation used to tune hyperparameters for the various models in an inner cross‐validation loop. Prediction results for each of the models (AUC, sensitivity, specificity) are gathered in an outer loop using leave‐one‐person‐out cross‐validation. This approach allows us to maximize sample size for training and testing by setting aside only one subject for testing in each iteration. Moreover, because the cross‐validation is blocked by person, we account for the nested structure of repeated observations. The performance metrics generated by this procedure are averaged across all participants and days.

After gathering these results, we selected the best model with respect to both performance and parsimony. We then examined feature importance to assess the relative contributions of the various predictors to predicting passive SI. In the case of regularized regression models, we applied the Least Absolute Shrinkage and Selection Operator (LASSO) penalty, which produces sparser results by forcing the coefficients of unselected variables to zero. This variable selection yields sets of coefficients that are more interpretable than elastic net regression.

As noted, we considered the importance of baseline features by training two versions of each model (elastic net, joint, and random forest). However, it is possible that baseline predictors play a relatively larger role in the days immediately following discharge because fewer days with longitudinal data are available. To better understand how this dynamic evolves over time, we examined the performance of our best model on each day, rather than averaged over the entire study period. Moreover, we constructed baseline‐day interaction terms by dividing the value of each baseline variable by the number of days the participant had been in the study, resulting in features that were more heavily weighted earlier in the study. By comparing the accuracy of both versions (with baseline features plus baseline‐day interactions and without any baseline features) on each day, we determined whether models that include baseline information have an advantage during the initial days following discharge.

## 3 Results

Active and passive SI were present in 631 (28.9%) and 729 (33.3%) person‐days, respectively. Sixty‐six out of 77 participants (85.71%) reported at least one instance of passive SI. A total of 30 (4.7%) instances of active SI occurred in the absence of any passive SI, whereas 128 (17.5%) instances of passive SI occurred in the absence of any active SI. Results for model AUC are shown in Table 2. The persistence model yielded an AUC of 0.832 while other approaches all achieved AUC close to 0.9. There was little difference in the performance of elastic net, joint, and random forest models. Table 3 illustrates a similar finding with respect to sensitivity and specificity, though the random forest procedure seems to be a less sensitive model. Furthermore, the inclusion of baseline features had a negligible effect on the predictive ability of our models. Descriptive statistics for the raw daily predictors are reported in Data S2, and the corresponding correlation matrix is presented in Data S3.

**TABLE 2:** Model performance (AUC; 95% CI).

Model	Baseline	AUC
Persistence	—	0.832
Elastic Net	Yes	0.901 [0.898, 0.903]
	No	0.901 [0.899, 0.903]
Joint	Yes	0.897 [0.895, 0.899]
	No	0.900 [0.899, 0.902]
Random Forest	Yes	0.896 [0.894, 0.898]
	No	0.898 [0.895, 0.899]

**TABLE 3:** Model performance (sensitivity and specificity; 95% CI).

Model	Baseline	Sensitivity	Specificity
Elastic Net	Yes	0.778 [0.771, 0.787]	0.885 [0.878, 0.891]
	No	0.793 [0.784, 0.801]	0.866 [0.859, 0.872]
Joint	Yes	0.780 [0.769, 0.790]	0.875 [0.866, 0.882]
	No	0.784 [0.775, 0.791]	0.873 [0.866, 0.883]
Random Forest	Yes	0.714 [0.699, 0.727]	0.909 [0.897, 0.920]
	No	0.764 [0.745, 0.782]	0.868 [0.848, 0.884]

**FIGURE 1:**
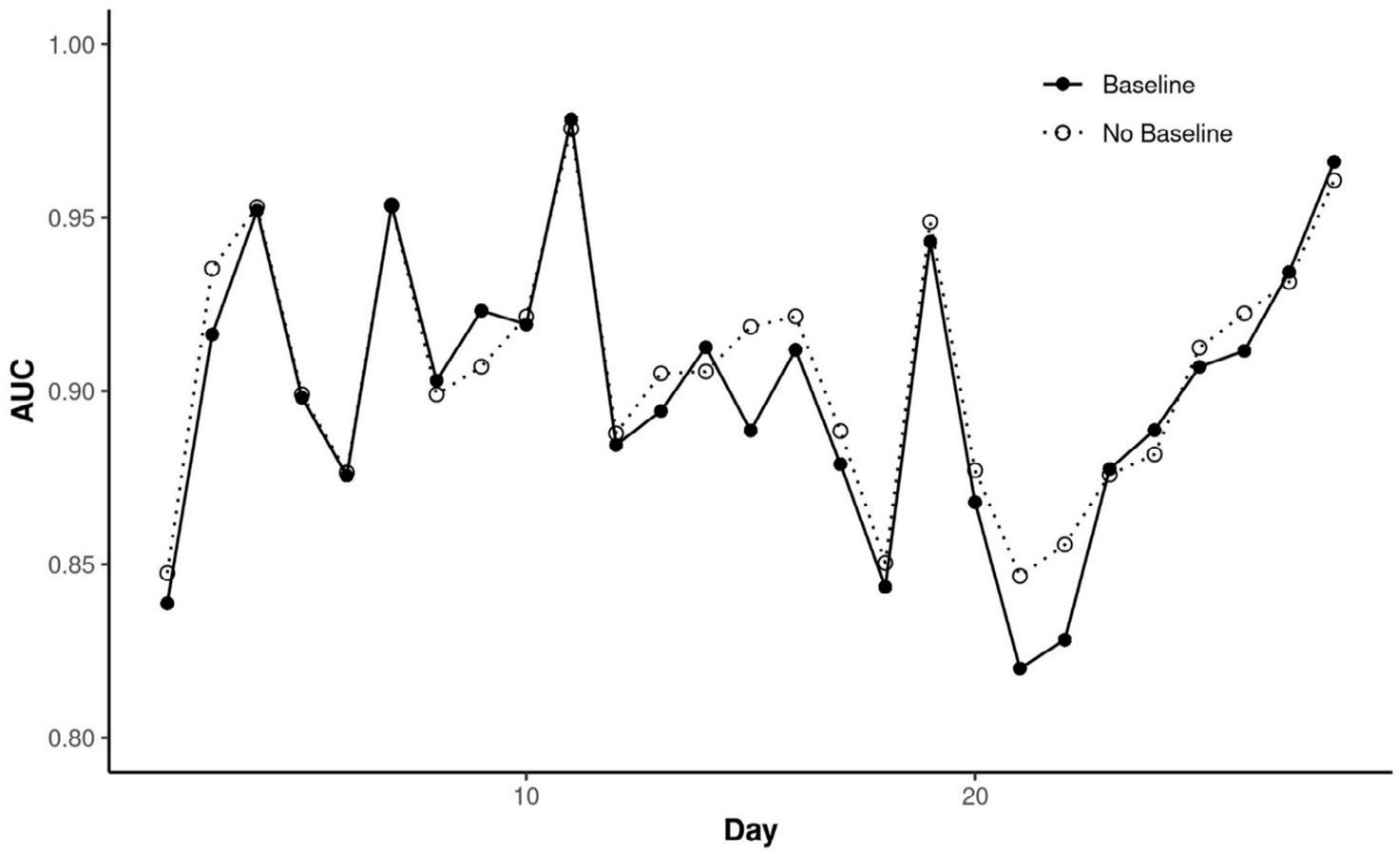
Elastic net AUC by day with and without baseline features.

Figure 1 shows the elastic net AUC for each day in the study when baseline variables are included/excluded as predictors. Despite weighting the baseline predictors higher in the beginning of the study when fewer days are available to construct cumulative time‐varying features, their inclusion offers no boost to AUC. This trend also occurred in joint and random forest models, shown in Data S4.

**FIGURE 2:**
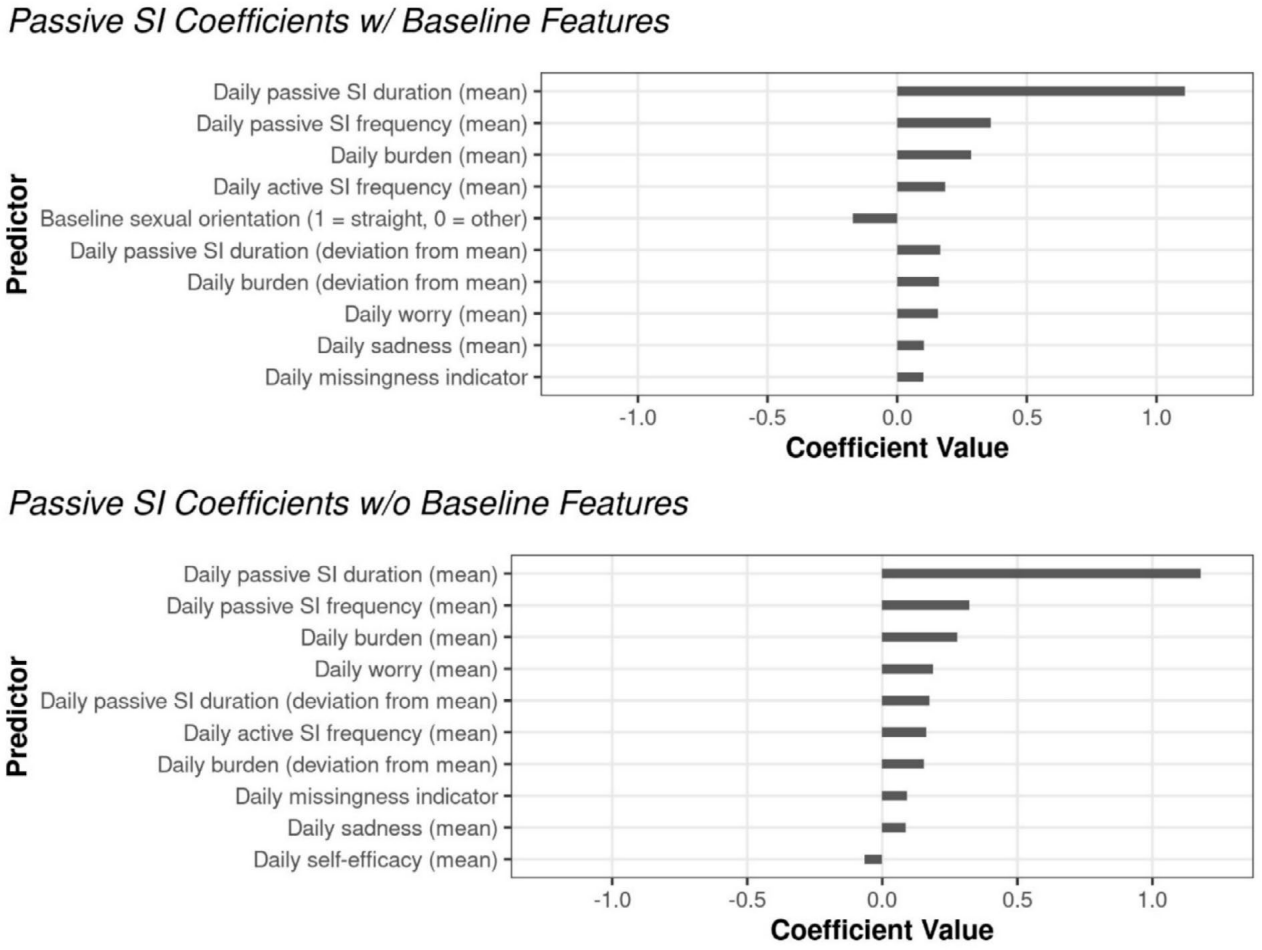
Feature importance (LASSO regression coefficients).

Figure 2 shows the LASSO coefficient estimates for the ten selected predictors with the largest magnitude estimates. Because the predictors were standardized, all coefficients are on the same scale. The most informative predictors are very similar across the two models with and without baseline features. To assess general model fit, an ordinary least squares regression model was estimated on the same data used to compute the LASSO coefficients (i.e., no baseline‐day interactions), yielding *R* ^2^ = 0.5171 with baseline predictors and *R* ^2^ = 0.5115 without baseline predictors.

## 4 Discussion

This secondary data analysis represents, to our knowledge, the first initiative aiming to predict the occurrence of next‐day passive SI using machine learning. The results reported herein establish a preliminary benchmark for the accuracy of such predictions among recently discharged adolescent inpatients. Repeated observations of time‐varying constructs were more useful for identifying next‐day thoughts of death than initial characteristics measured at baseline, even during the first few days post‐discharge when time‐varying data were still relatively scarce. Among the time‐varying features examined, facets of passive SI (duration, frequency) were the strongest predictors.

Across machine learning models, with varying degrees of complexity, findings demonstrated that next‐day passive SI can be predicted with good overall predictive accuracy (AUC = 0.896–0.901). Rather than comparing accuracy against random chance or base rate, we included a strong comparison condition (the persistence model) to assess the viability of our predictive models. All the tested models outperformed the persistence model. The accuracy of the best‐performing model (AUC = 0.901) corresponds to a very strong effect size (*d* = 1.8; Salgado 2018). Of note, the best model was a relatively simple Elastic Net regression. More complex nonlinear or joint models did not improve this approach. These results are consistent with previous machine learning studies and systematic reviews which found that algorithmic complexity does not necessarily confer a significant advantage (Horwitz et al. 2023; Jacobucci et al. 2021; Burke et al. 2019). Simpler machine learning models, such as those based on linear regression techniques, may be preferable because they are easier to interpret than highly parameterized “black box” algorithms. Enhancing both interpretability and clinical applicability is vital for integrating these models into routine practice (Nordin et al. 2022).

The current results are comparable to the accuracy of predicting active SI in youth (Czyz, Koo, et al. 2021) and marginally better than predicting active SI in adults (Czyz et al. 2023). Passive SI is often assumed to be part of a gradient of SI that is less severe and subsumed by the presence of active SI. For example, this is reflected in the hierarchical SI structure of the widely‐used Columbia Suicide Severity Rating Scale (Posner et al. 2011). Nevertheless, predicting passive SI on its own is an important goal. There are significant risks associated with passive SI (Liu et al. 2020) and some individuals who attempt suicide report never experiencing active ideation (Wastler et al. 2021). In this study, passive SI was more frequent than active SI and more likely to occur in isolation. Thus, the sole focus on active SI might risk missing the opportunity to anticipate and prevent this prevalent phenomenon that contributes to risk of suicidal behavior.

The field of suicide prevention is increasingly recognizing the importance of considering both initial or trait‐like characteristics and time‐varying indices of risk (measured repeatedly), with a corresponding surge in studies employing intensive longitudinal methods to explore between‐ and within‐person patterns (e.g., Bagge and Borges 2017). However, the differential importance of initial versus time‐varying constructs remains relatively unexplored, particularly in real‐world contexts. The current study design highlights a unique challenge: The first week after an inpatient admission is a high‐risk period, yet it is precisely the period with the fewest repeated observations of risk indices. This challenge is often described as the “cold start” problem in machine learning research. Personalized multitask learning is a promising new approach that leverages both between‐person and within‐person features to predict short‐term outcomes. One way to accomplish this is to train unique predictive models on time‐varying features within clusters of individuals that have similar profiles of baseline risk factors (see Taylor et al. 2020). The current study represents an initial inquiry into the feasibility of this approach for predicting SI. If baseline constructs have the potential to improve predictions through personalization, then we would expect the inclusion of those variables to contribute some useful information to our basic predictive models. Ultimately, this was not the case. Incorporating these initial characteristics did not improve the prediction of next‐day passive SI above and beyond daily features. This held true during the initial days of the study period when fewer daily observations were available, even after weighting the model inputs to prioritize baseline features during those days. This suggests that clustering on the initial characteristics assessed in this study may be less useful. In contrast, given the predominance of dynamic features, models based on intensely‐measured temporal profiles (Czyz et al. 2022) may be fruitful.

Many theoretical constructs are purportedly related to the occurrence of SI. Yet, despite the inclusion of well‐known risk factors, specific facets of passive SI itself were the most useful features for predicting next‐day passive SI. Among these, the average duration of passive SI across the previous 7 days was by far the strongest predictor, followed by the average frequency. Focusing on duration, this is consistent with previous research that has highlighted duration of SI as a critical indicator of next‐day active SI (Czyz, Koo, et al. 2021), a predictor of future psychiatric emergencies (Gipson et al. 2014), and a marker associated with higher odds of future suicide attempts (Miranda et al. 2014), although duration in this earlier work largely referred to active SI. Prolonged duration of passive SI may reflect sustained engagement with suicidal thoughts over time. One possible interpretation is that duration indexes suicide‐specific rumination (Rogers and Joiner 2018a, 2018b), characterized by repetitive, intrusive, and hard‐to‐disengage thinking about one's suicidal thoughts. However, it is important to note that this form of rumination was not directly assessed in the study, and this interpretation remains speculative. Nonetheless, the prominence of duration as a predictor provides support for interventions designed to manage enduring SI by increasing cognitive flexibility and awareness of alternate solutions, such as problem‐solving therapy (Eskin et al. 2007; Gustavson et al. 2016), or mindfulness‐based approaches targeting rumination, such as mindfulness‐based cognitive therapy (Sipe and Eisendrath 2012).

Beyond facets of passive SI, the observed pattern of predictor importance offers several theory‐relevant insights, although a comprehensive evaluation of leading ideation‐to‐action suicide theories at short timescales was beyond the scope of the present study. In contrast to facets of passive SI, several theory‐driven constructs (e.g., 7‐day daily averages of hopelessness and connectedness) did not emerge among the strongest predictors of next‐day passive SI. One possible interpretation is that leading theories of SI may be less informative for short‐term prediction of passive SI and instead more relevant for explaining the development and maintenance of suicide risk over longer intervals (e.g., months or years). This interpretation is consistent with emerging evidence from intensive longitudinal studies testing the IPTS. For instance, when controlling for concurrent SI, Kraiss et al. (2024) found that none of the IPTS constructs predicted next time‐point active and passive SI in about half of their sample, and Kleiman et al. (2017) similarly reported that none of the IPTS constructs predicted next time‐point active SI. Together, this work suggests that while these constructs remain theoretically meaningful, their predictive utility for near‐term change of suicide risk may be limited. Notably, the 7‐day daily average of burdensomeness emerged among the top‐ranked predictors–following duration and frequency of passive SI–whereas other theory‐derived constructs did not. This finding is consistent with prior work illustrating that perceived burdensomeness is often a more robust correlate of SI than thwarted belongingness (Chu et al. 2017), including in intensive longitudinal studies examining both passive and active SI (Hallensleben et al. 2019). In summary, though the present study was optimized for prediction rather than theory testing, the findings provide theory‐relevant insights into short‐term passive SI. Future work explicitly designed to examine differences between passive and active SI across different timescales may further clarify how theory‐driven constructs operate across the continuum of SI.

Considering the above results together, tracking daily ratings of the frequency and duration of passive SI is an important consideration for predicting near‐term occurrence of passive SI. Adding model complexity, jointly modeling active SI, and including initial baseline features did not incrementally improve prediction accuracy. Future researchers faced with decisions about what constructs to assess and when could focus on intensive longitudinal assessment of facets of passive SI versus more extended symptom batteries at baseline. It is tempting to delve into more extensive history taking and evaluation to obtain potentially useful data that can make the job of preventing suicide easier. Our inclusion of a relatively modest set of nine baseline predictors did not prove useful towards this end. This is consistent with the findings of Franklin et al. (2017) who demonstrated that comprehensive inventories of risk factors have yielded very little clinical utility. The current study balances their recommendation to shift focus away from risk factors towards machine learning algorithms, while still exploring the possibility of meaningful contribution from these baseline variables.

Practically, the current findings strongly suggest that clinicians should prioritize consistent repeated assessment of facets of passive SI. Mobile health technologies may provide an avenue for the monitoring of SI at scale. Prior work suggests that clinicians generally view additional monitoring and feedback about patients' suicide risk during high‐risk periods as somewhat positive and minimally burdensome (Glenn et al. 2022). Related qualitative work has also reported generally positive attitudes, interest, and conditional acceptability of automated suicide risk assessment models (Bentley et al. 2022; Dayal et al. 2025; Yarborough et al. 2022). Notably, much of this work has focused on prototypes of single suicide risk estimates at one point of care, limiting conclusions about ongoing clinician engagement with real‐time and dynamically updated risk information. Nevertheless, these findings emphasize the importance of ongoing evaluation and collaboration with clinicians to determine how repeated screening and monitoring of passive SI can be meaningfully integrated into routine care. These findings are particularly relevant to informing personalized prevention strategies that rely on forecasting near‐term states of risk or vulnerability to deploy timely support, such as Just‐in‐Time Adaptive Interventions (Coppersmith et al. 2022; Nahum‐Shani et al. 2017). As demonstrated in this study, simpler models may be sufficient when constructing prediction models that inform these prevention techniques. In practice, such forecasts can be used to prompt brief, supportive responses to users, such as reminders to use coping strategies or crisis resources during elevated periods of risk.

The current study possesses many strengths. The study involved a high‐risk adolescent sample and was conducted during a critical high‐risk window for suicidal ideation and behavior. The combination of sample characteristics, timing, and repeated observations presents a unique opportunity to explore questions about the relative importance of trait‐ and state‐level predictors and how prediction performance evolves over time. The data were subjected to a rigorous blocked and nested cross‐validation process, increasing confidence that the models will generalize to out‐of‐sample predictions and that performance metrics faithfully approximate real‐world accuracy. Yet, there are also important limitations. Although the predictive models were carefully cross‐validated, replication in an independent data set would provide the strongest evidence of generalizability. The sample was relatively small (*N* = 77) for machine learning approaches, mostly demographically and culturally homogeneous, and participants were all recruited from a single site; future work should evaluate the generalizability of these findings in larger, more diverse settings and in less acute clinical settings beyond the immediate post‐discharge period.

Additional methodological limitations include a high missingness rate of passive SI duration (82%) and the subsequent use of imputation by regression onto passive SI frequency. Although these variables were highly correlated and the imputation model was statistically sound, the large number of duration values that were imputed—rather than directly observed—may have reduced variability. As such, findings pertaining to the duration construct should be considered preliminary and interpreted carefully. Key constructs, namely facets of passive and active SI, were assessed using single items adapted from the C‐SSRS to reduce participant burden and maximize feasibility of compliance with daily assessments. Although prior studies have similarly adapted the C‐SSRS to reflect EMA‐specific recall periods (Kivelä et al. 2022), and there has been growing psychometric support for single‐item indicators of SI in EMA research (Forkmann et al. 2018; Hendley et al. 2025; Spangenberg et al. 2025), EMA‐specific validation of adapted C‐SSRS items has not yet been established. Future research should formally evaluate EMA‐adapted single‐item measures of SI and examine how their performance compares with validated multi‐item assessments, which may offer more psychometric precision but at the cost of increased participant burden. Additionally, daily self‐reports may have been susceptible to recall bias, even over short timeframes.

Finally, we note that the prediction target in this study was a form of ideation, not suicidal behavior. Any recommendations or insights gleaned from these results should be interpreted with that distinction in mind. Passive SI may represent one pathway to suicidal behavior (Wastler et al. 2021) and is worthy of attention, but predicting actual behavior remains a paramount, yet elusive goal.

In conclusion, this study highlights the potential of real‐time monitoring and machine learning to forecast short‐term risk of passive SI with high predictive accuracy, thereby augmenting suicide prevention efforts. Our findings suggest that time‐varying indicators, particularly frequency and duration of passive SI, may serve as near‐term predictors of passive SI and notably outperform initial baseline characteristics. However, the predictive utility of passive SI duration should be interpreted with some caution, given the high proportion of statistically derived values in the current sample. Future work is needed to confirm the predictive utility of time‐varying predictors of passive SI, including directly measured passive SI duration, in larger and more diverse populations of individuals at risk. Finally, future work should evaluate how these findings may be implemented into real‐time personalized suicide prevention strategies.

## Author Contributions

**Luke Francisco:** formal analysis, visualization, methodology, writing – original draft, writing – review and editing. **Ewa Czyz:** conceptualization, funding acquisition, investigation, supervision, writing – review and editing. **Ambuj Tewari:** conceptualization, formal analysis, investigation, methodology, writing – review and editing, supervision. **Megan Chen:** conceptualization, writing – original draft, writing – review and editing. **Shane Kentopp:** conceptualization, formal analysis, investigation, methodology, writing – original draft, writing – review and editing.

## Ethics Statement

This study and procedures were approved by the Michigan Medicine Institutional Review Board (HUM00129173).

## Consent

All participants gave informed consent before participation.

## Conflicts of Interest

The authors declare no conflicts of interest.

## Supporting information


**Data S1:** Complete case analysis.

**Data S2:** Table B.1. Descriptive statistics for raw daily predictor variables.

**Data S3:** Correlation matrix of daily predictors.

**Data S4:** Figure D.1. Joint model AUC by day.

## Data Availability

The data that support the findings of this study are available from the corresponding author upon reasonable request.
