# Supplementary material for: Predicting Next‐Day Passive Suicidal Ideation in At‐Risk Youth
Source: Suicide Life Threat Behav. 2026 Jul 2;56(4):e70124. doi: 10.1111/sltb.70124 (PMC13324972; doi:10.1111/sltb.70124)

**Online Supplement A: Complete Case Analysis**

To gauge the robustness of our methods to our selected imputation procedure, we perform all prior analyses on a set of complete cases. Our complete case analysis consisted of 1345 daily observations across 77 subjects for models without baseline features and 1335 daily observations across 75 subjects for models with baseline features. The results of this procedure are shown in the tables and figures below. Overall, it seems our results are similar to those obtained on the full data set.

| **Table A.1**  *Model Performance (AUC)* | | |  |
| --- | --- | --- | --- |
| Model | Baseline | AUC |  |
| Elastic Net | Yes | 0.905 |  |
|  | No | 0.907 |  |
| Joint | Yes | 0.903 |  |
|  | No | 0.906 |  |
| Random Forest | Yes | 0.908 |  |
|  | No | 0.910 |  |
| **Table A.2**  *Model Performance (Sensitivity and Specificity)* | | |  |
| Model | Baseline | Sensitivity | Specificity |
| Elastic Net | Yes | 0.784 | 0.876 |
|  | No | 0.799 | 0.864 |
| Joint | Yes | 0.796 | 0.867 |
|  | No | 0.809 | 0.868 |
| Random Forest | Yes | 0.716 | 0.908 |
|  | No | 0.795 | 0.868 |

**Figure A.1**

*Daily AUC for the Elastic Net Model With and Without Baseline Features*


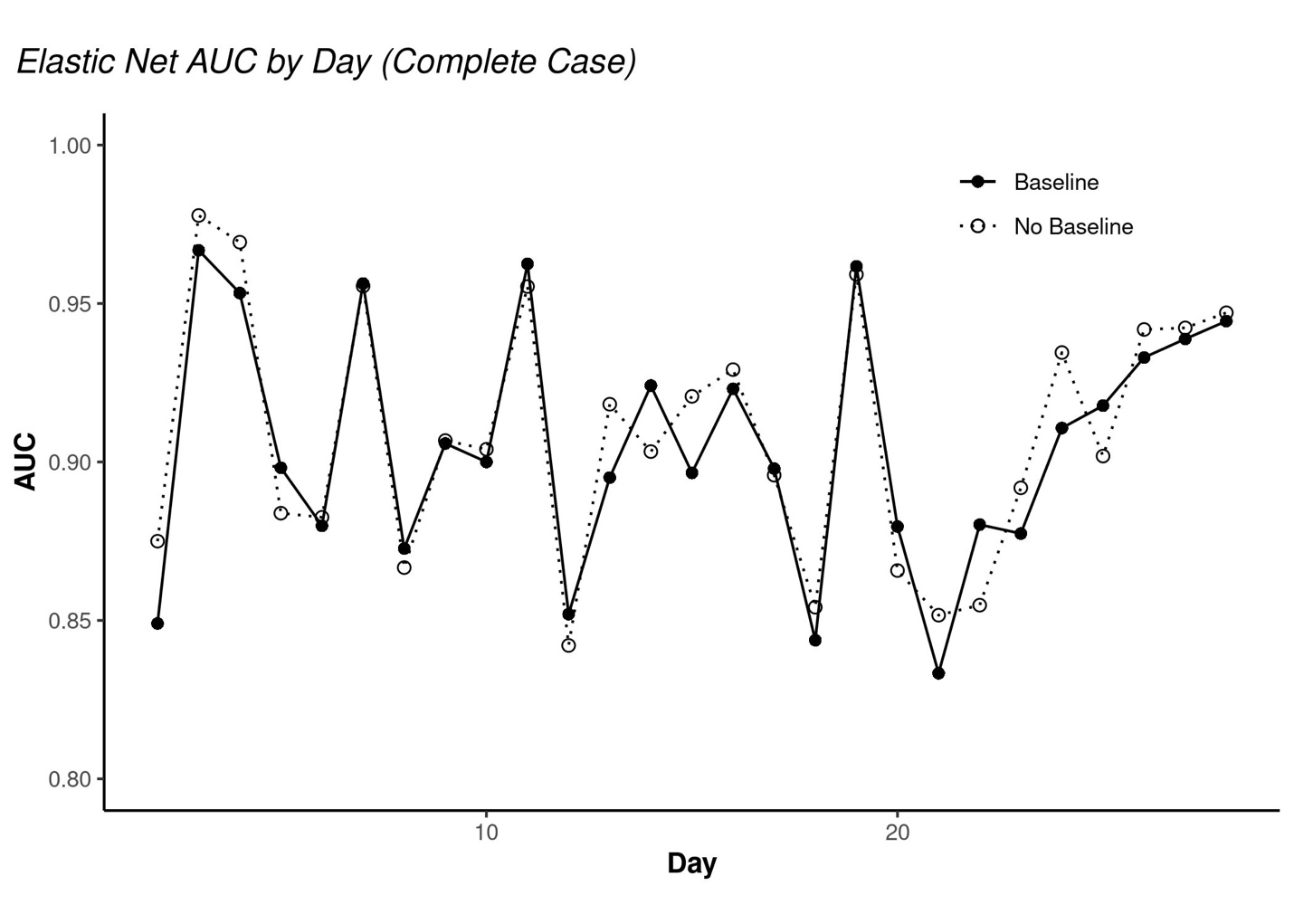


**Figure A.2**

*Daily AUC for the Joint Model With and Without Baseline Features*


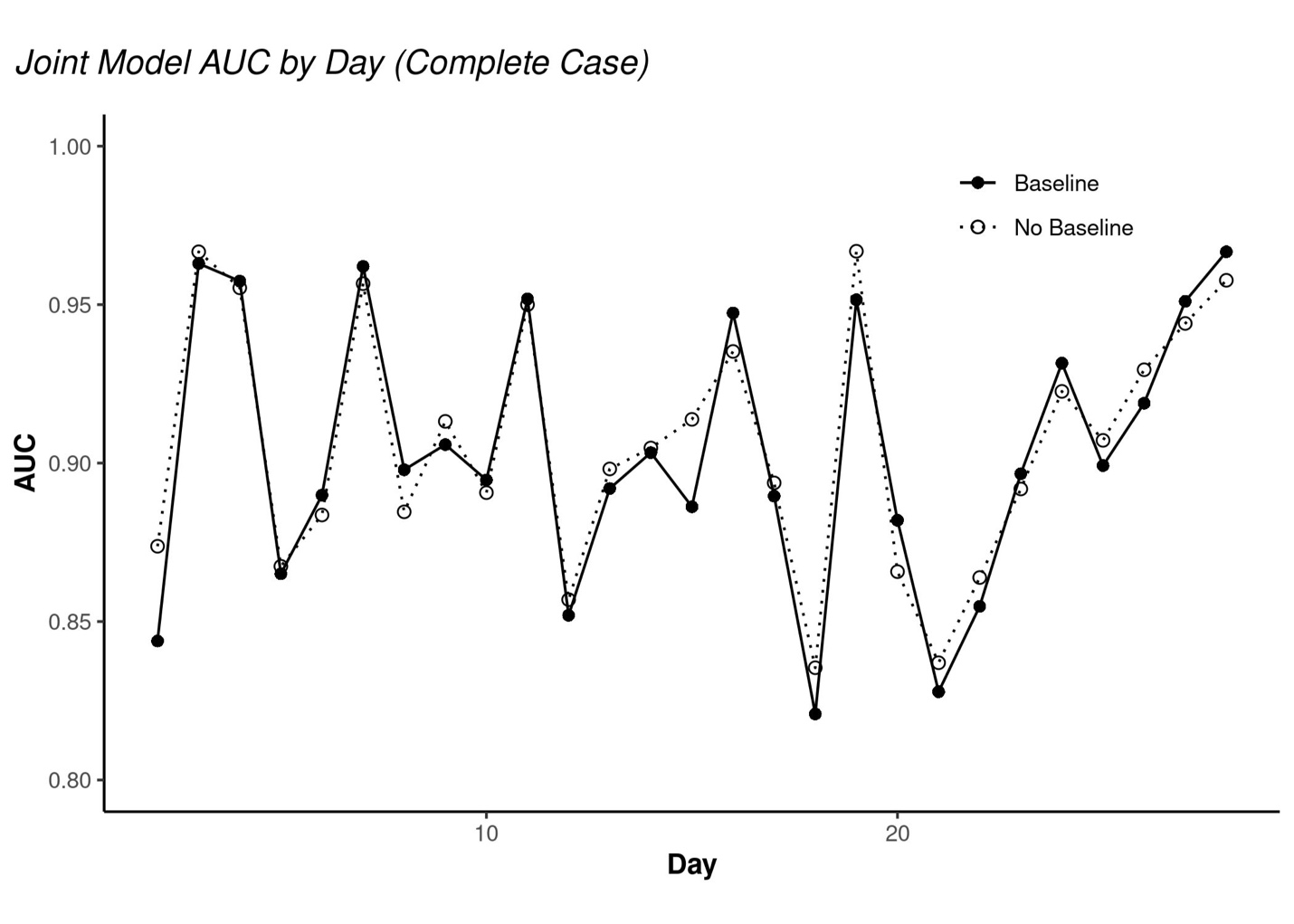


**Figure A.3**

*Daily AUC for the Random Forest Model With and Without Baseline Features*


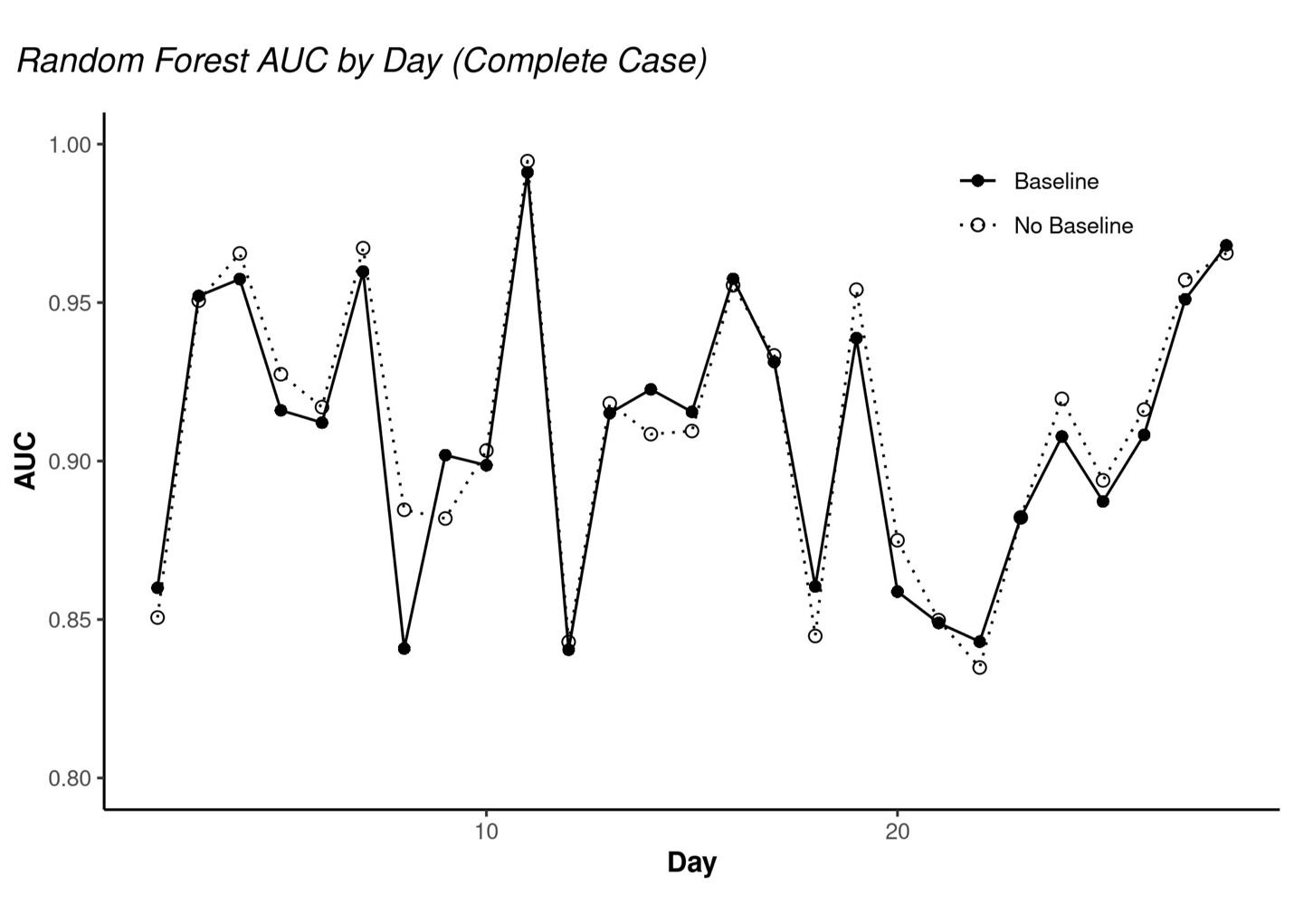


**Figure A.4**

*Top 10 Largest Magnitude Coefficients from the LASSO Procedure*


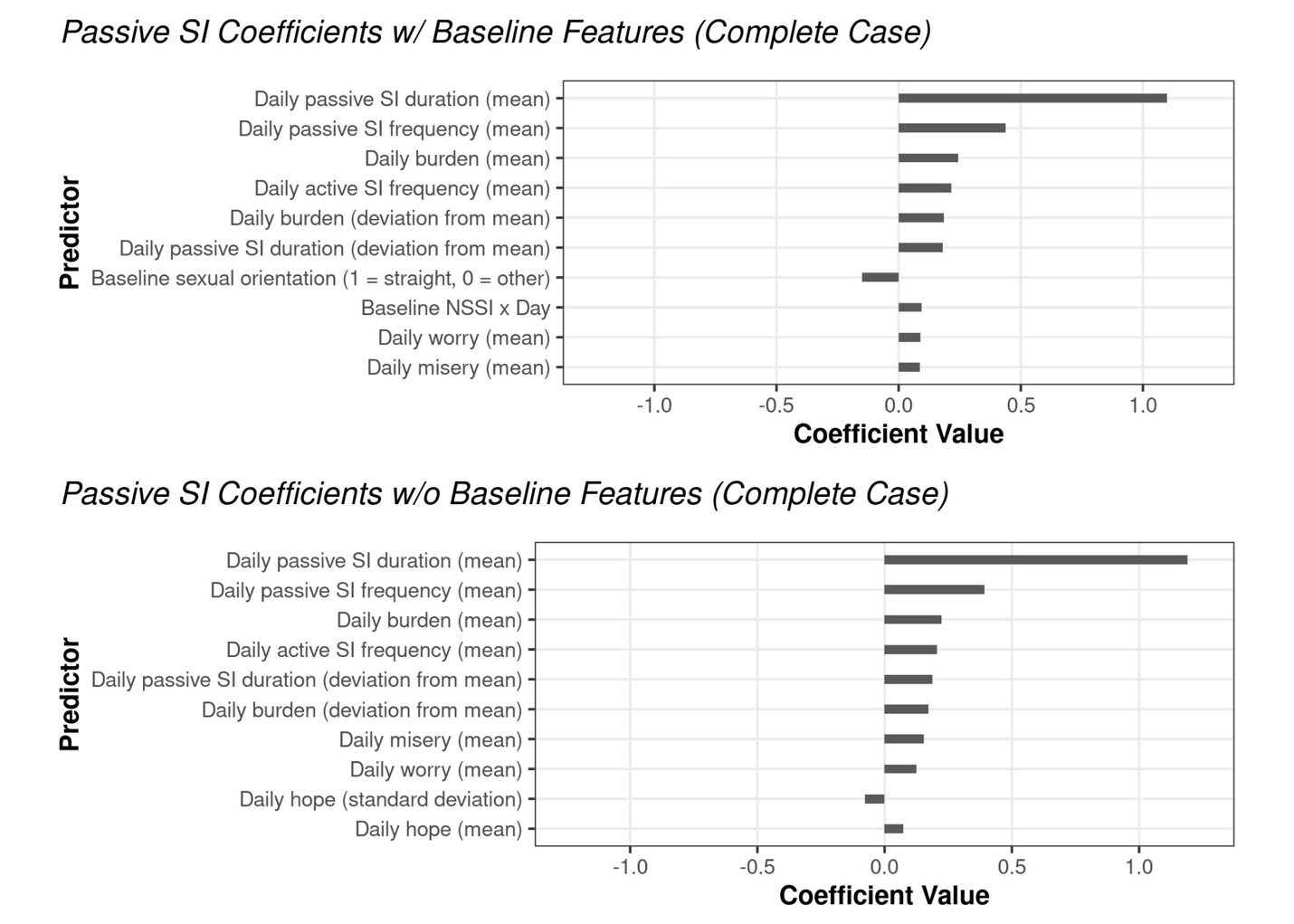


**Figure A.5**

*10 Most Significant Coefficients Predicting Passive SI in the Mixed-Effects Model*


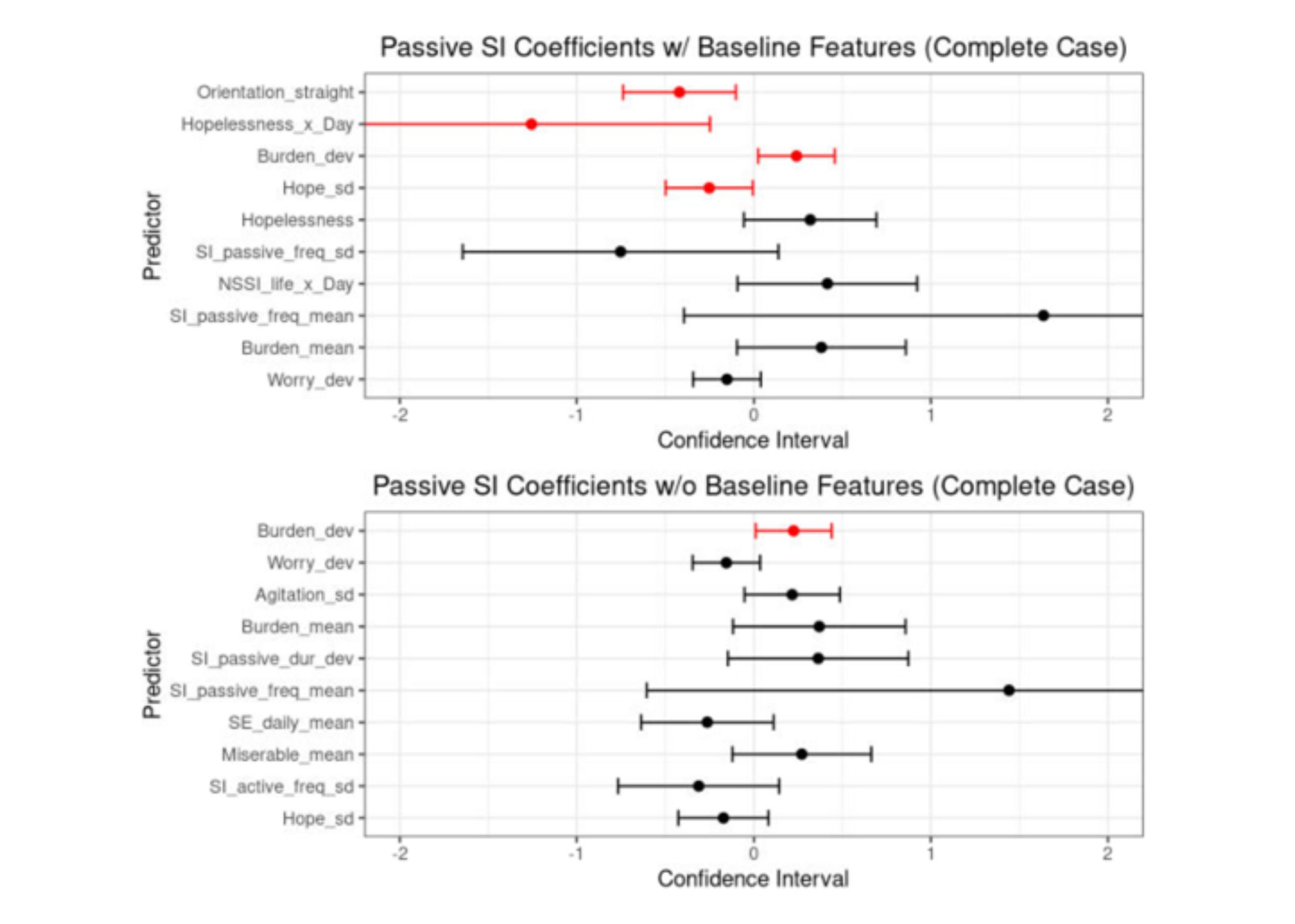

Supplement: Supplementary file 1 — Data S1: Complete case analysis. [file SLTB-56-0-s004.docx]
