# Supplementary material for: Predicting Next‐Day Passive Suicidal Ideation in At‐Risk Youth
Source: Suicide Life Threat Behav. 2026 Jul 2;56(4):e70124. doi: 10.1111/sltb.70124 (PMC13324972; doi:10.1111/sltb.70124)
Supplement: Supplementary file 2 — Data S2: Table B.1. Descriptive statistics for raw daily predictor variables. [file SLTB-56-0-s003.docx]

**Online Supplement B**

**Table B.1**
*Descriptive Statistics for Raw Daily Predictor Variables*

| Predictor | Mean (SD) [ICC] |
| --- | --- |
| SI passive frequency | 0.97 (1.26) [0.67] |
| SI passive duration | 1.12 (1.44) [0.66] |
| SI active frequency | 0.77 (1.11) [0.62] |
| SI active duration | 0.90 (1.32) [0.60] |
| Closeness | 4.76 (1.52) [0.54] |
| Self-efficacy | 7.91 (2.51) [0.66] |
| Burden | 2.86 (1.86) [0.62] |
| Hope | 2.01 (0.91) [0.58] |
| Agitation | 2.95 (2.21) [0.62] |
| Worry | 3.41 (1.91) [0.45] |
| Rumination | 3.15 (1.79) [0.47] |
| Sad | 2.51 (1.26) [0.50] |
| Miserable | 2.10 (1.16) [0.50] |
| Happy | 3.55 (1.04) [0.47] |

*Note.* Values reflect descriptive statistics for raw daily features, not constructed variables used in primary analyses. Means are reported first, followed by standard deviations in parentheses and intra-class correlations (ICCs) in brackets.
