# Supplementary figures and images for: Predicting Next‐Day Passive Suicidal Ideation in At‐Risk Youth
Source: Suicide Life Threat Behav. 2026 Jul 2;56(4):e70124. doi: 10.1111/sltb.70124 (PMC13324972; doi:10.1111/sltb.70124)

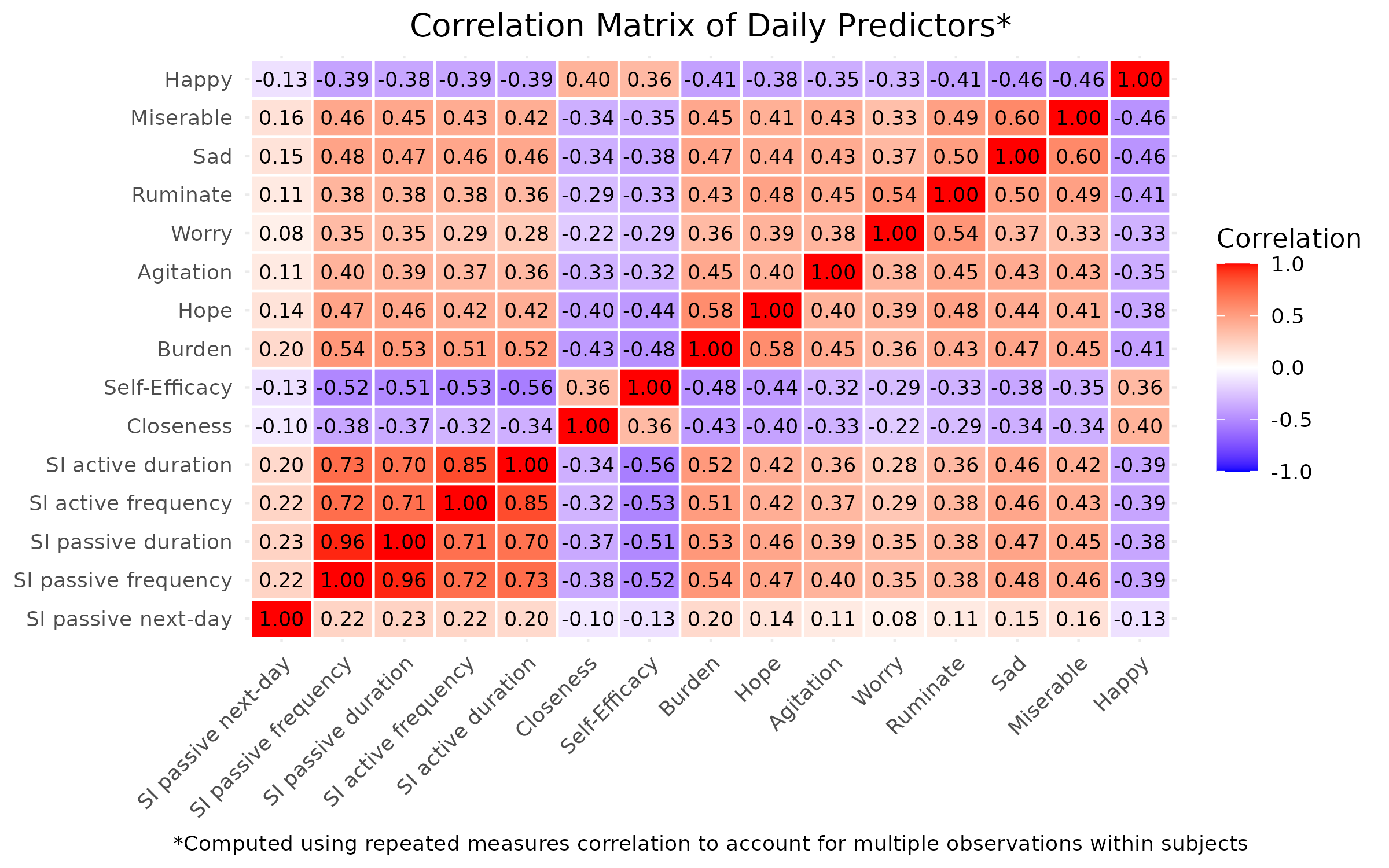

Supplement: Supplementary file 3 — Data S3: Correlation matrix of daily predictors. [file SLTB-56-0-s001.png]

**Online Supplement D**

**Figure D.1**

*Joint Model AUC by Day*
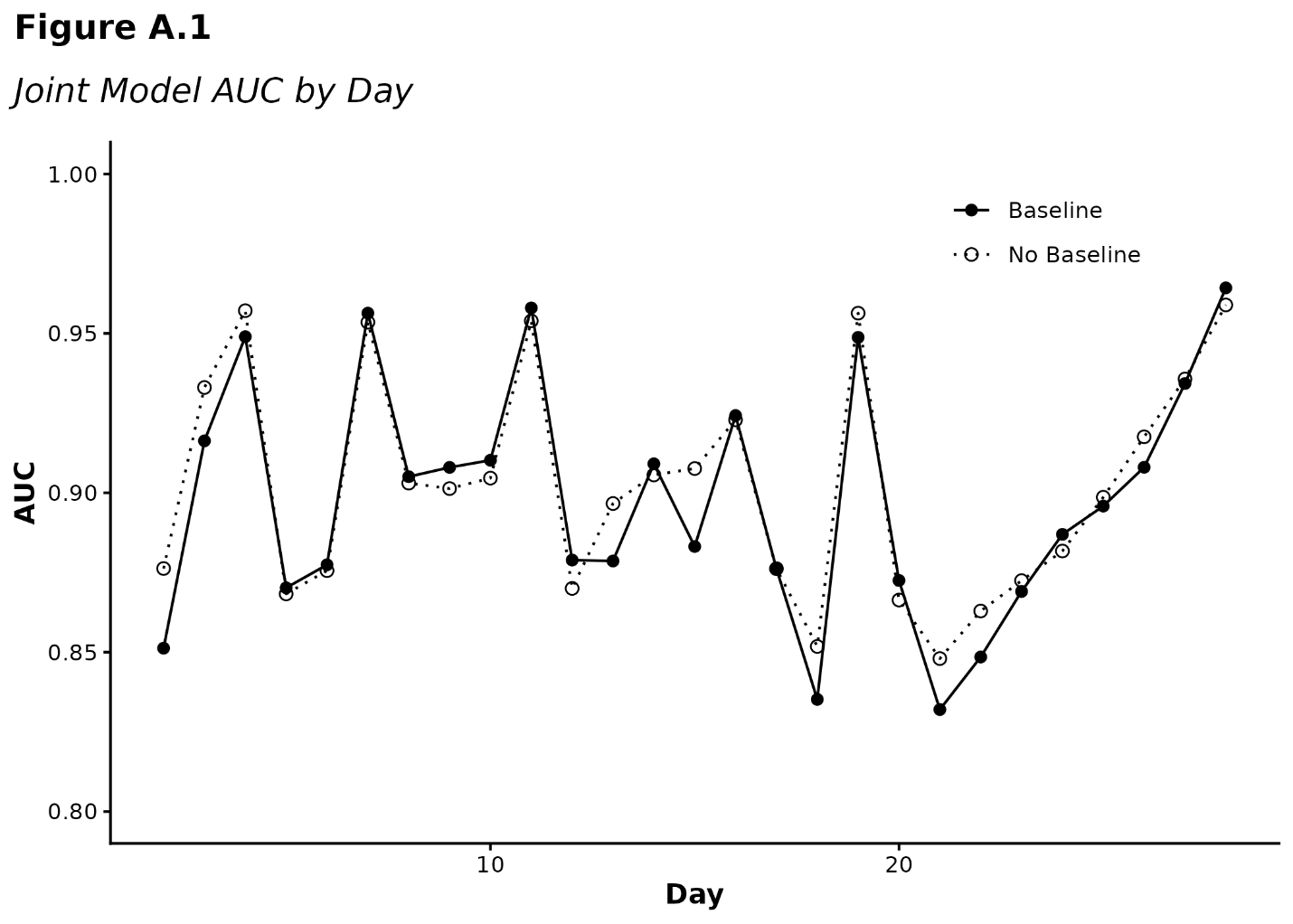


**Figure D.2**

*Random Forest AUC by Day*


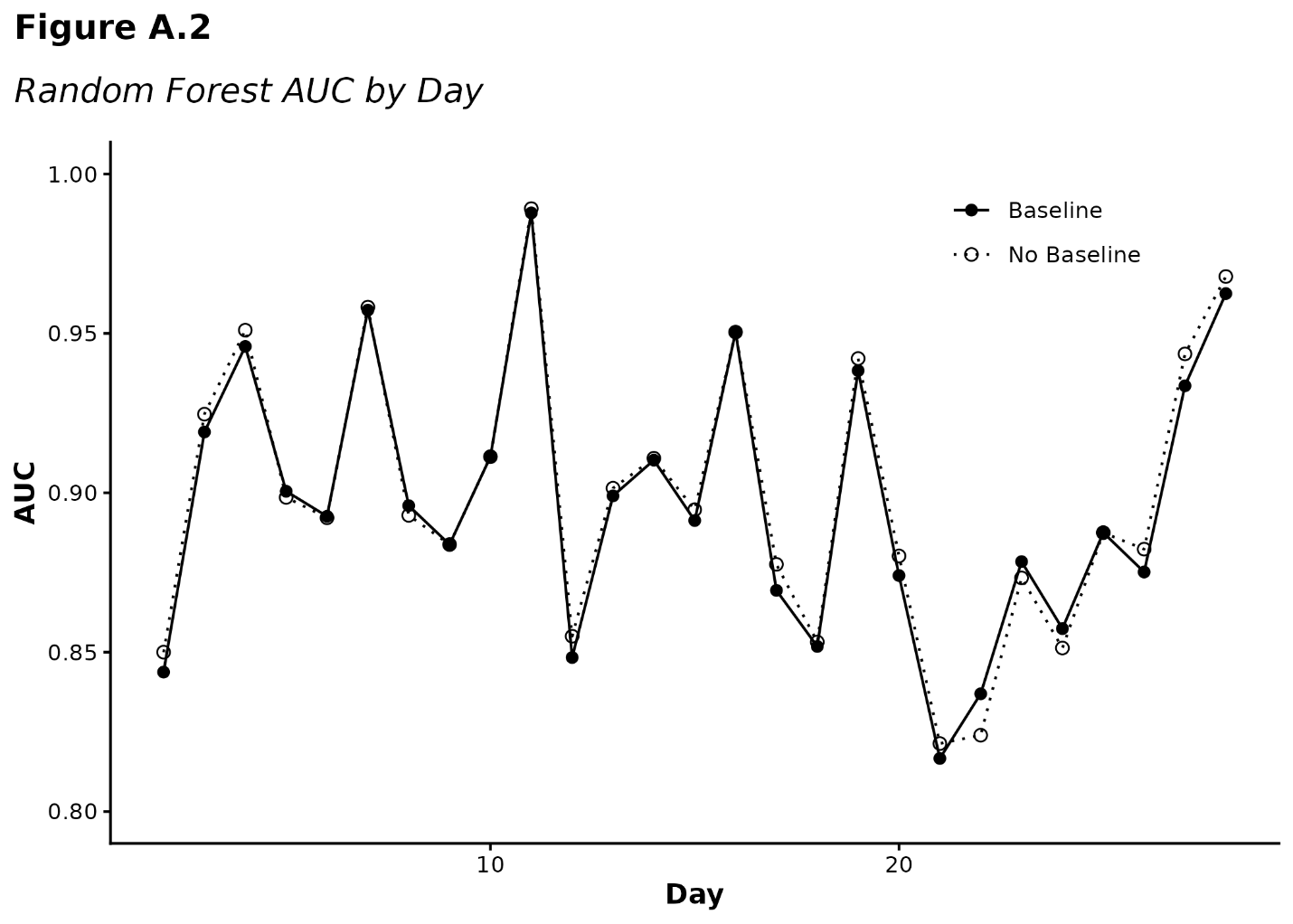

Supplement: Supplementary file 4 — Data S4: Figure D.1. Joint model AUC by day. [file SLTB-56-0-s002.docx]
